# The association of liver enzymes with diabetes mellitus risk in different obesity subgroups: A population-based study

**DOI:** 10.3389/fendo.2022.961762

**Published:** 2022-10-13

**Authors:** Dinghao Zheng, Xiaoyun Zhang, Lili You, Feng Li, Diaozhu Lin, Kan Sun, Meng Ren, Li Yan, Wei Wang

**Affiliations:** Department of Endocrinology, Sun Yat-sen Memorial Hospital, Sun Yat-sen University, Guangzhou, China

**Keywords:** GGT, AST, ALT, diabetes mellitus, obesity

## Abstract

**Background:**

Numerous observational studies have shown that liver enzymes correlated with diabetes mellitus (DM) risk significantly, but limited studies showed whether different obesity subgroups present the same correlation. Our objective was to evaluate the association of liver enzymes with DM risk in different obesity subgroups based on a middle-aged Chinese population.

**Methods:**

We conducted a population-based cross-sectional study and surveyed 9,916 people aged 40 years and above. A two-slope linear regression model was used to analyze the cutoff points of obesity in DM risk. Restricted cubic splines were used to analyze the correlation between liver enzymes and DM risk in different obesity categories. The odds ratios and 95% confidence intervals (CIs) were calculated using the logistic regression model.

**Results:**

The cutoff points of body mass index (BMI) and waist circumference were 30.55 kg/m^2^ and 98.99 cm for DM risk, respectively. The serum gamma-glutamyl transferase (GGT) concentration was positively correlated with DM risk in the subgroups with waist circumference <98.99 cm [OR = 1.04, 95% CI (1.03–1.05)], BMI <30.55 kg/m^2^ [OR = 1.04, 95% CI (1.03–1.05)], and BMI ≥30.55 kg/m^2^ [OR = 1.18, 95% CI (1.04–1.39)], but not in the subgroup with waist circumference ≥98.99 cm. Alanine aminotransferase (ALT) and aspartate aminotransferase (AST) concentrations have no significant correlation with the risk of diabetes in all groups.

**Conclusion:**

The results showed that serum GGT concentration was correlated with DM risk but not with AST or ALT in the middle-aged population. However, the correlation disappeared when waist circumference was over 98.99 cm, and serum GGT concentration had a limited value for DM risk in waist circumference over 98.99 cm.

## Introduction

According to the International Diabetic Federation diabetes atlas (10th edition, 2021), the global prevalence of diabetes has reached 10.5% and is expected to rise to 11.3% in 2030 and 12.2% in 2045. Approximately 6.7 million people (20–79 years old) died of diabetes or its complications in 2021, accounting for approximately 12.2% of all deaths worldwide (1, 2). According to an epidemiological survey, the prevalence of diabetes mellitus in mainland China was 12.8% in 2017, indicating that diabetes mellitus has become an important public health problem in the world, especially in China (3). Therefore, identifying the high-risk population of diabetes is important to solving the public diabetes burden.

Numerous observational studies indicated that liver enzymes such as aspartate aminotransferase (AST), alanine aminotransferase (ALT), and gamma-glutamyl transferase (GGT) were positively correlated with the risk of diabetes mellitus and metabolic syndrome, especially serum GGT (4–11). The liver plays an important role in maintaining the homeostasis of glucose metabolism, and liver enzyme concentrations are positively correlated with liver damage. Serum ALT and AST are released from the injured hepatocytes. GGT widely exists on the surface of cell membranes and is a key participant in the metabolism of glutathione. Glutathione is an important cellular antioxidant, which is closely related to inflammation and oxidative stress in tissues (12, 13), and oxidative stress and inflammation play an important role in the development of insulin resistance (14).

Obesity refers to a state that is obviously overweight, caused by the excessive accumulation of body fat (15). Obesity can promote global inflammation and peripheral insulin resistance, increasing the prevalence of various metabolic diseases, such as diabetes and non-alcoholic fatty liver disease (16, 17). According to a 2015–2017 national cross-sectional study in China, the incidence of overweight and obesity is 30.1% and 11.9%, respectively, and the proportion of the population with a body mass index (BMI) ≥30 kg/m^2^ is 6.3%. The prevalence of diabetes mellitus (DM) in BMI <25 kg/m^2^, 25 kg/m^2^ ≤ BMI < 30 kg/m^2^, and BMI ≥30 kg/m^2^ is 8.8%, 13.8%, and 20.1%, respectively (3).

To better assess the correlation between liver enzymes and DM risk, obesity classification should be considered. However, limited studies showed whether obesity plays a role in the correlation between liver enzymes and the risk of diabetes mellitus. So, we conducted a large population cross-sectional study in Guangzhou, China, to clarify the relationship and provided guidance for identifying a high-risk population of diabetes mellitus.

## Method

### Study populations

**Figure 1:**
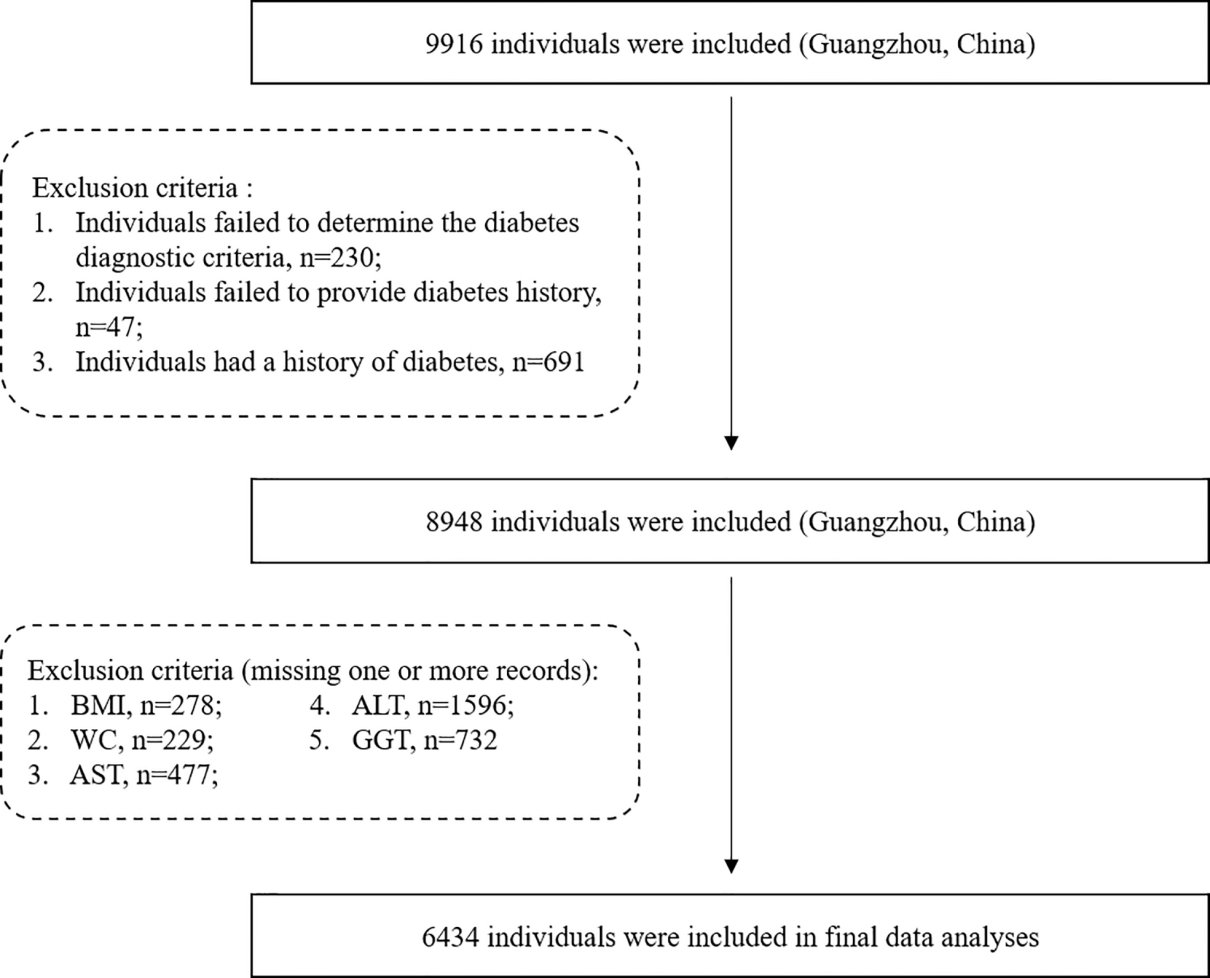
Study population flowchart.

From June to November 2011, we performed a community-based cross-sectional research in Guangzhou, China. The participants in this study were selected from the Risk Evaluation of Cancers in Chinese Diabetic Individuals: A longitudinal (REACTION) research project, which has established a multicenter prospective observational study to evaluate chronic diseases in the Chinese population (18). The study population, design, and protocol have been previously described (19). Briefly, through inspection notices or home visits, a total of 10,104 residents aged 40 or over were invited to participate. A total of 9,916 individuals accepted to participate in the poll after signing the permission form, and the participation rate was 98.1%. Among these participants, individuals who failed to provide BMI (*n* = 278), waist circumference (WC) (*n* = 229), liver enzymes (*n* = 2,127, one or more among the AST, ALT, GGT), blood glucose (*n* = 230), and diabetes history information (*n* = 47) were excluded, and individuals with diabetes history were also excluded (*n* = 691) (**Figure 1**). Finally, a total of 6,434 qualified individuals were included in the final data analysis.

The research protocol has been approved by the Institutional Review Committee of Sun Yat-sen Memorial Hospital affiliated to Sun Yat-Sen University and complies with the principles of the Declaration of Helsinki II. Each participant gave written informed consent before data were collected.

### Measurements

We collected information about lifestyle factors, sociodemographic characteristics, education information, marital status, and family history of diabetes using standard questionnaires. Lifestyle factors include smoking and drinking. Smoking or drinking habits are classified as “never,” “current” (smokers or drinks regularly in the past 6 months), or “never” (stops smoking or drinking for more than 6 months).

All participants used standard procedures to complete the anthropometric measurements with the assistance of trained personnel. With automatic electronic equipment (OMRON, Omron Company, China), blood pressure measurements were performed three consecutive times by the same observer at 5-min intervals. The analysis was performed using the average of three blood pressure measurements. Participants wore light clothing and had no shoes, and their height and weight were measured to be within 0.1 cm and 0.1 kg, respectively. BMI was computed by multiplying body weight (kg) by height (square meter) (kg/m^2^). WC was measured at the level of the umbilical cord when the participant is standing and at the end of a mild exhalation.

After an overnight fast for at least 10 h, a venous blood sample was collected for laboratory testing. Measurement of fasting blood glucose (FPG), fasting serum insulin, total cholesterol (TC), triglycerides (TG), high-density lipoprotein cholesterol (HDL-C), low-density lipoprotein cholesterol (LDL-C), GGT, AST, and ALT was performed using an automatic analyzer (Beckman CX-7 Biochemical Autoanalyzer, Brea, CA, USA). Hemoglobin A1c (HbA1c) was evaluated by high-performance liquid chromatography (Bio-Rad, Hercules, CA, USA). Diabetes is diagnosed according to the 1999 World Health Organization’s diagnostic criteria, including fasting blood glucose greater than or equal to 7.0 mmol/L and/or OGTT 2 h greater than or equal to 11.1 mmol/L.

### Statistical analysis

Continuous variables were presented as means ± standard deviation (SD). Skewed variables were presented as medians (interquartile ranges). Categorical variables were expressed as proportions. Differences among groups were tested by one-way ANOVA, and *post-hoc* comparisons were performed by using Bonferroni correction. Comparisons between categorical variables were performed with the *χ* ^2^ test.

We performed the two-slope regression model to determine the relationship between BMI or WC and the risk of diabetes to analyze the cutoff points for DM risk.

Linear regression and logistic regression were performed to calculate the odds ratios (ORs) of diabetes and 95% confidence intervals (95% CIs) after adjusting for age, gender, BMI, SBP, TG, and HDL. Restricted cubic splines were performed to visualize the shape of the dose–response association among the AST, ALT, GGT, and odds ratio of diabetes, respectively. All statistical analyses were performed using RStudio version 3.6.1. A two-tailed *p <*0.05 was considered statistically significant.

## Results

### Clinical characteristics of the study population

The clinical characteristics of the study population are shown in **Table 1**. The mean age of the diabetes group versus the non-diabetes group was 57.6 (7.00) vs. 54.7 (6.68). The diabetes group also had higher BMI, WC, DBP, SBP, CHOL, TG, and LDL and lower HDL (all *p* for trend <0.001). In addition, compared with the non-diabetes group, the diabetes group had higher serum GGT levels (24.0 vs. 19.0 U/L, *p* < 0.001) and higher serum ALT levels (13.0 vs. 12.0 U/L, *p* < 0.001), but with no significant difference for AST levels (19.0 vs. 18.0 U/L, *p* = 0.163).

**Table 1:** Characteristics of the study population.

Characteristics	Group		
	Non-diabetes	Diabetes	*p*
Male, *n* (%)	1,511 (27.2%)	238 (27.1%)	0.989
Age, mean (SD)	54.7 (6.68)	57.6 (7.00)	< 0.001
Status of marriage, *n* (%)	4,987 (90.3%)	772 (88.6%)	0.046
Elementary school and below, *n* (%)	564 (10.4%)	145 (17.0%)	< 0.001
Smoking, *n* (%)	514 (9.38%)	73 (8.48%)	0.622
Drinking, *n* (%)	154 (2.82%)	27 (3.13%)	0.280
Family history of diabetes, *n* (%)	853 (15.6%)	187 (21.8%)	< 0.001
Height, mean (SD)	158 (7.20)	158 (6.74)	0.007
Weight, mean (SD)	58.1 (8.54)	60.5 (8.34)	< 0.001
BMI, mean (SD)	23.2 (2.83)	24.4 (2.92)	< 0.001
WC, mean (SD)	80.3 (8.40)	84.3 (8.39)	< 0.001
HC, mean (SD)	93.4 (6.13)	94.7 (6.19)	< 0.001
SBP, mean (SD)	125 (16.0)	133 (16.8)	< 0.001
DBP, mean (SD)	74.8 (9.77)	77.6 (9.77)	< 0.001
HR, mean (SD)	80.4 (10.3)	82.9 (10.7)	< 0.001
CHOL, mean (SD)	5.25 (1.06)	5.44 (1.10)	< 0.001
TG, median (IQR)	1.19 [0.89; 1.62]	1.49 [1.10; 1.97]	< 0.001
HDL, mean (SD)	1.36 (0.33)	1.27 (0.32)	< 0.001
LDL, mean (SD)	3.18 (0.84)	3.31 (0.90)	< 0.001
AST, median (IQR)	18.0 [16.0; 21.0]	19.0 [16.0; 22.0]	0.163
ALT, median (IQR)	12.0 [9.00; 16.0]	13.0 [9.00; 17.0]	< 0.001
GGT, median (IQR)	19.0 [14.0; 25.0]	24.0 [18.0; 31.0]	< 0.001

### The effect of BMI and WC on the risk of diabetes

**Figure 2:**
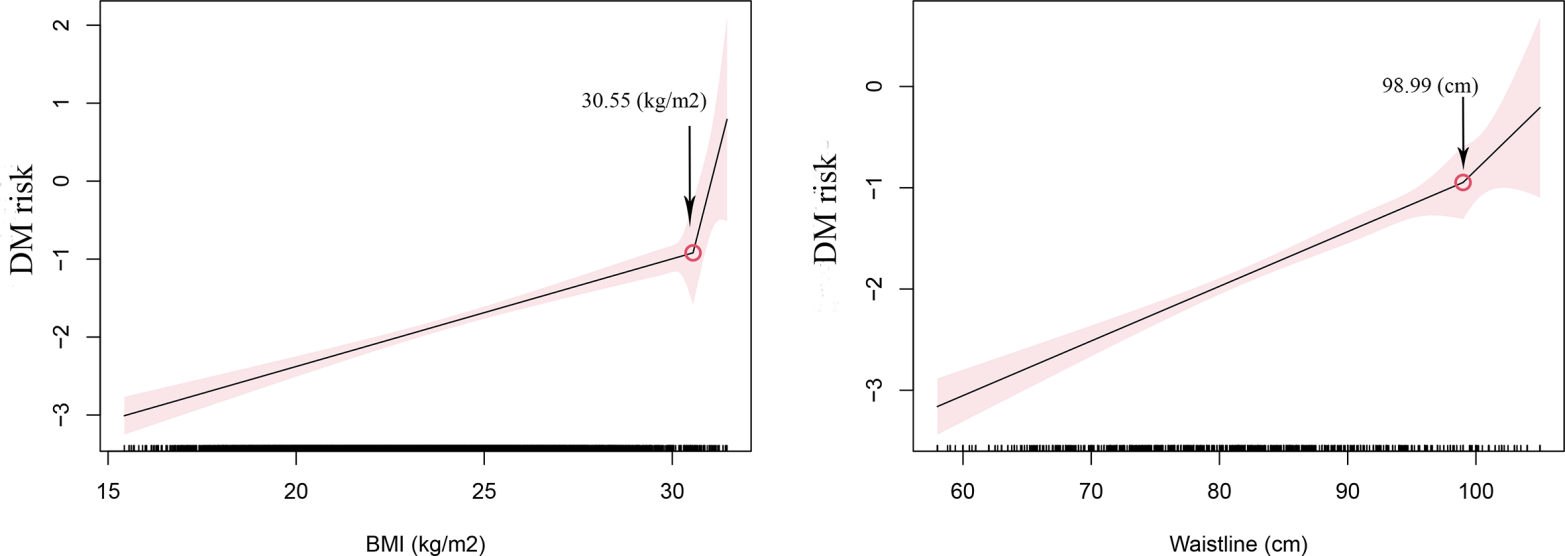
The effect of body mass index (BMI) and waist circumference (WC) on the risk of diabetes.

In order to explore the appropriate cutoff points of BMI and WC for the risk of diabetes, we performed the two-slope regression model to visualize the association of BMI or WC on the risk of diabetes. The cutoff points for BMI and WC were 30.55 kg/m^2^ and 98.99 cm, respectively (**Figure 2**). The results showed that when BMI or WC exceeds 30.55 kg/m^2^ or 98.99 cm, respectively, the risk of diabetes will increase significantly.

### The curve correlation between liver enzymes and DM risk

**Figure 3:**
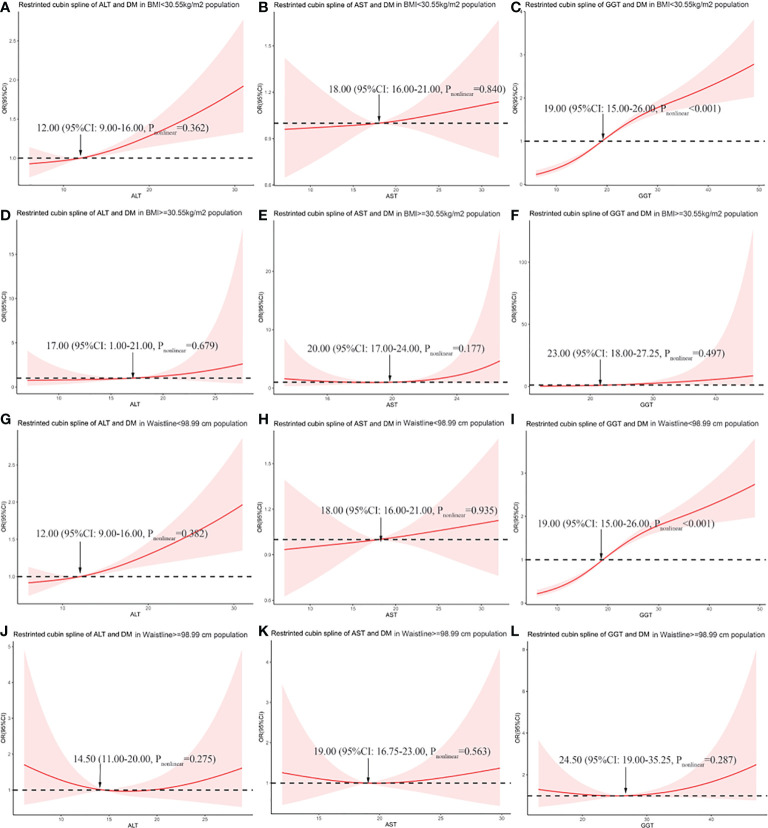
The association between liver enzymes and the risk of diabetes in different BMI and WC subgroups. **(A)** Association between alanine aminotransferase (ALT) and odds ratio (OR) in the subgroup with BMI <30.55 kg/m^2^. **(B)** Association between aspartate aminotransferase (AST) and OR in the subgroup with BMI <30.55 kg/m^2^. **(C)** Association between gamma-glutamyl transferase (GGT) and OR in the subgroup with BMI <30.55 kg/m^2^. **(D)** Association between ALT and OR in the subgroup with BMI ≥30.55 kg/m^2^. **(E)** Association between AST and OR in the subgroup with BMI ≥30.55 kg/m^2^. **(F)** Association between GGT and OR in the subgroup with BMI ≥30.55 kg/m^2^. **(G)** Association between ALT and OR in the subgroup with WC <98.99 cm. **(H)** Association between AST and OR in the subgroup with WC <98.99 cm. **(I)** Association between GGT and OR in the subgroup with WC <98.99 cm. **(J)** Association between ALT and OR in the subgroup with WC ≥98.99 cm. **(K)** Association between AST and OR in the subgroup with WC ≥98.99 cm. **(L)** Association between GGT and OR in the subgroup with WC ≥98.99 cm.

We explored the correlation between liver enzymes and the risk of DM in different BMI and WC subgroups, according to the cutoff point above, using restricted cubic spline graphs. The results showed that in the population with BMI <30.55 kg/m^2^, ALT and AST have no curvilinear correlation with the risk of DM (*p* = 0.362 and *p* = 0.840, respectively), and GGT has a curvilinear correlation with the risk of DM (*p* < 0.001) (**Figures 3A–C**). In the population with BMI ≥30.55 kg/m^2^, liver enzymes have no curvilinear correlation with the risk of DM (all *p* > 0.05) (**Figures 3D–F**). According to WC, in the population with WC <98.99 cm, our study found that ALT and AST have no curvilinear correlation with the risk of DM (*p* = 0.382 and *p* = 0.935, respectively), and GGT has a curve correlation with the risk of DM (*p* < 0.001) (**Figures 3G–I**). It is particularly worth noting that in the population with a WC ≥98.99 cm, although the statistical analysis of the curve relationship has no significance (all *p* > 0.05), the restricted cubic spline graph showed a U-shaped relationship between liver enzymes and DM risk (**Figures 3J–L**).

### The association of liver enzymes with DM risk in different BMI subgroups

In our study, we grouped the subjects according to the cutoff point of BMI and explored the relationship between liver enzymes and the risk of diabetes in different groups. The results of the study showed that only GGT levels have a significant correlation with the DM risk (**Table 2**). After adjusting for confounding factors, the OR (95% CI) is 1.04 (1.03–1.05, *p* < 0.001) in the population with BMI <30.55 kg/m^2^, and the OR (95% CI) is 1.18 (1.04–1.39, *p* < 0.024) in the population with BMI ≥30.55 kg/m^2^. As for AST and ALT, after adjusting for confounding factors, the ORs (95% CI) were 0.98 (0.96–1.01, *p* = 0.094) and 1.00 (0.99–1.02, *p* = 0.573) in the subgroup with BMI <30.55 kg/m^2^, respectively. Moreover, the ORs (95% CI) were 1.16 (0.98–1.39, *p* = 0.095) and 1.05 (0.94–1.20, *p* = 0.412) in the subgroup with BMI ≥30.55 kg/m^2^.

**Table 2:** The linear regression model of the relationship between liver enzymes and DM risk in BMI subgroups.

Liver enzymes category	Fold change (95% CI) of DM risk per unit increase in liver enzymes			
	Unadjusted model		Adjusted model^a^	
BMI < 30.55 kg/m^2^ (*n* = 6,386)				
AST	1.01 (0.99–1.02)	*p* = 0.398	0.98 (0.96–1.01)	*p* = 0.094
ALT	1.03 (1.01–1.04)	*p* < 0.001	1.00 (0.99–1.02)	*p* = 0.573
GGT	1.05 (1.04–1.06)	*p* < 0.001	1.04 (1.03–1.05)	***p* < 0.001**
BMI ≥ 30.55 kg/m^2^ (*n* = 48)				
AST	1.08 (0.95–1.24)	*p* = 0.263	1.16 (0.98–1.39)	*p* = 0.095
ALT	1.06 (0.97–1.16)	*p* = 0.202	1.05 (0.94–1.20)	*p* = 0.412
GGT	1.13 (1.05–1.26)	*p* = 0.001	1.18 (1.04–1.39)	***p* = 0.024**

a Covariates in the adjusted model: gender, age, BMI, SBP, TG, and HDL. Bold value are statistically significant.

### The association of liver enzymes with DM risk in different WC subgroups

Subjects were grouped according to the cutoff points of WC. We explored the association between liver enzymes and DM in different WC subgroups (**Table 3**). After adjusting for confounding factors, the results showed that GGT only has a positive correlation with DM risk in the population with WC <98.99 cm, and the OR (95% CI) is 1.04 (1.03–1.05, *p* < 0.001). In the population with WC ≥98.99 cm, the serum GGT has no significant correlation with DM risk, and the OR (95% CI) is 1.02 (0.98–1.06, *p* = 0.365). As for ASL and ALT, they had no significant correlation with DM risk in both WC subgroups.

**Table 3:** Regression model of the relationship between liver enzymes and DM risk in WC subgroups.

Liver enzymes category	Fold change (95% CI) of DM risk per unit or class increase in liver enzymes			
	Unadjusted model		Adjusted model^a^	
Waistline < 98.99cm^b^ (*n* = 6,290)				
AST	1.01 (0.99–1.02)	*p* = 0.369	0.99 (0.97–1.01)	*p* = 0.161
ALT	1.03 (1.02–1.04)	*p* < 0.001	1.01 (0.99–1.02)	*p* = 0.388
GGT	1.05 (1.04–1.06)	*p* < 0.001	1.04 (1.03–1.05)	***p* < 0.001**
Waistline ≥ 98.99 cm^b^ (*n* = 144)				
AST	1.01 (0.94–1.08)	*p* = 0.891	1.00 (0.92–1.09)	*p* = 0.958
ALT^b^	1.00 (0.95–1.05)	*p* = 0.984	0.98 (0.92–1.04)	*p* = 0.558
GGT^b^	1.02 (0.99–1.05)	*p* = 0.260	1.02 (0.98–1.06)	*p* = 0.365
Waistline ≥ 98.99 cm^c^				
AST				
≤16.75	1.42 (0.58–3.40)	*p* = 0.436	1.29 (0.47–3.47)	*p* = 0.618
16.75–23.00	1.00	–	1.00	–
≥23.00	1.97 (0.85–4.57)	*p* = 0.111	2.16 (0.80–5.88)	*p* = 0.128
ALT				
≤11.00	1.57 (0.68–3.65)	*p* = 0.288	1.78 (0.67–4.82)	*p* = 0.249
11.00–20.00	1.00	–	1.00	–
≥20.00	1.67 (0.69–4.07)	*p* = 0.254	1.29 (0.46–3.57)	*p* = 0.627
GGT				
≤19.00	1.09 (0.45–2.55)	*p* = 0.845	1.67 (0.60–4.72)	*p* = 0.326
19.00–35.25	1.00	–	1.00	–
≥32.25	1.56 (0.66–3.65)	*p* = 0.305	2.07 (0.75–5.78)	*p* = 0.127

a Covariates in the adjusted model: gender, age, BMI, SBP, TG, and HDL.

b Linear regression models for liver enzymes.

c Logistic regression models for the liver enzyme category. Bold value are statistically significant.

According to the restricted cubic spline graph (**Figures 3J–L**), in the population with WC ≥98.99 cm, liver enzymes show a U-shaped relationship with DM risk. Therefore, we divided them into three categories according to the distribution of liver enzymes. AST was divided into three categories according to 16.75 and 23.00 U/L, and ALT was divided according to 11.00 and 20.00 U/L. GGT was divided based on 19.00 and 32.25 U/L (**Table 3**). For all liver enzymes, the middle range group was used as the reference group (the ALT reference category was 16.75–23.00 U/L, the AST reference category was 11.00–20.00 U/L, and the GGT reference category was 19.00–32.25 U/L). The results showed that liver enzymes have no correlation with the risk of diabetes significantly in the population with WC ≥98.99 cm (all *p* > 0.05).

## Discussion

In this manuscript, we focused on the relationship between liver enzymes and DM risk in different obesity subgroups in a cross-sectional study. We got the cutoff points of BMI and WC according to the two-slope linear regression model, and restricted cubic splines were used to analyze the correlation between liver enzymes and DM risk in different classes of the obese population divided according to cutoff points. Our results showed that only serum GGT level was correlated with DM risk instead of ALT and AST. It is worth noting that the correlation disappeared in the subgroup with WC ≥98.99 cm.

For obesity categories, previous studies conventionally grouped individuals according to the WHO criterion or percentile (20, 21). Our obesity classifications were not based on these criteria, considering that the criteria cannot reflect DM risk accurately. We conducted a two-slope regression model to find out the cutoff point where the DM risk increased faster. The cutoff points were 98.99 cm and 30.55 kg/m^2^, respectively. Interestingly, the BMI cutoff point was similar to the WHO obesity classification (BMI ≥ 30 kg/m^2^); however, the Chinese always use the Asian obesity classification (BMI > 28 kg/m^2^). Moreover, the WC cutoff point was much higher than the abdominal obesity classification (WC > 90 cm for men and WC > 85 cm for women). We used the common criteria as the cutoff point to repeat the analysis, and the results showed a significant association between GGT and DM risk in all groups (**Table S1**). The results implied that different regional populations with different ages may need more special criteria for DM risk and other obesity complications.

Our study showed that after grouping by BMI or WC, there was no significant correlation between ALT or AST and DM risk, and only GGT was correlated in some subgroups. The results were consistent with previous studies (4, 22–24). The results suggested that GGT levels were more significant for evaluating DM risk than other liver enzymes. However, there were other studies that showed conflicting results. A population study in Europe showed that ALT was significantly correlated with impaired glucose tolerance (IGT) but not with GGT or AST (25), suggesting that ALT and prediabetes are closely related. However, the study had limitations. The sample size of that study was 157, and it only adjusted for age and gender. A population study in China showed that GGT levels were not significantly associated with HOMA-IR in non-alcoholic fatty liver disease patients (NAFLD). The study only included 212 patients. The sample size may not be powerful and the HOMA-IR was not equal to DM risk (26). Another prospective study in Korea that included 548 patients showed that GGT levels were associated with DM risk only in women but not in men. Our study population mainly consisted of women, which may lead to conflicting results (4). A Mendelian randomization study showed that genetically higher ALT was associated with a higher DM risk but not GGT (27). However, the Mendelian randomization study was based on genes, not considering environment function and compensation function, and we cannot exclude the possibility that GGT was associated with DM risk. GGT is a protein that exists widely on the cell membrane and is closely related to the metabolism of glutamate in cells. GGT regulates the level of oxidative stress in cells and tissues, which is closely related to diabetes mellitus risk (14). In this respect, GGT has a more reasonable metabolic relevance, which partly explains why GGT in liver enzymes has a more significant diabetes risk association than ALT or AST.

We observed that GGT was associated with DM risk in different subgroups, but this association disappeared in the group with WC ≥98.99 cm. The result showed that the association will be disturbed for severe abdominal obesity individuals. A 15,792 middle-aged community-based prospective cohort study in the USA showed that GGT was associated with DM risk, after adjusting for body mass index, WC, and other confounding factors (28). The result was inconsistent with ours to some extent, and for this reason, we cannot get the association in the group with WC ≥98.99 cm. The contradiction may rely on WC stratification. The possible mechanism underlying the phenomenon was unclear and may be proposed as follows. Severe abdominal obesity may be a confounding factor for GGT and DM risk, which was similar to other points (29). Abdominal obesity with increased visceral fat is closely related to systemic inflammation and oxidative stress, and abnormal oxidative stress usually leads to increased serum GGT concentrations (12). So, patients with severe abdominal obesity, who are at high DM risk, may be accompanied by elevated serum GGT concentration raised from abnormal oxidative stress and led to the confounding associations between GGT and DM. The other explanation was that the risk factors for the development of diabetes mellitus in the population with severe abdominal obesity were not similar in other groups. Furthermore, the risk of DM reflected by GGT may not play a major role in a population with severe abdominal obesity, and GGT will also show different associations with DM risk in a population with different pathophysiological states.

Our research also has certain limitations. The major limitation is that the data of the ultrasonic diagnosis of fatty liver were incomplete, considering that the liver enzymes were highly associated with fatty liver and we could not exclude the confounding factor. Secondly, our study has a cross-sectional design. Causal inferences could not be drawn between serum GGT and DM risk among different subgroups, so more in-depth prospective studies may be needed to prove it. Thirdly, in the subgroup of people with WC ≥98.99 cm, the disappearance of the relationship may lie in the sample size of the subgroups (*n* = 144), and we should get more samples to ensure the associations. Lastly, the population in this study consists of middle-aged individuals aged >40 years old in South China and cannot represent the younger population and the North China population. Furthermore, the population mainly consists of women, partially because we invited residents over the age of 40 years and women are predominant in this age range in China. The pathophysiological mechanism of this research still needs to be further clarified.

In summary, in this cross-sectional study of a large population, we found that increased serum GGT levels are correlated with the risk of diabetes, and showed different effects in subgroups with different BMI or WC values. Serum GGT may be a better reference marker for predicting the risk of diabetes mellitus than AST or ALT in clinical practice. Individuals with elevated liver enzymes, especially GGT, should be alert to the risk of diabetes mellitus. However, GGT has limitations on DM risk in a population with severe abdominal obesity. For this population, even elevated GGT cannot reflect the risk of diabetes mellitus, and other biomarkers should be considered.

## Conclusion

Our study suggests that serum GGT levels have greater reference significance than AST or ALT for the risk of diabetes in the middle-aged population. Moreover, GGT levels correlate with DM risk except for those with severe abdominal obesity. In clinical practice, GGT should be combined with WC to determine DM risk.

## Data availability statement

The raw data supporting the conclusions of this article will be made available by the authors, without undue reservation.

## Ethics statement

The studies involving human participants were reviewed and approved by Institutional Review Committee of Sun Yat-sen Memorial Hospital affiliated to Sun Yat-Sen University. The patients/participants provided their written informed consent to participate in this study.

## Author contributions

All authors listed have made a substantial, direct, and intellectual contribution to the work, and approved it for publication.

## Funding

This study was supported by grants from the National Science Foundation of China (U20A20352) and Guang Dong Clinical Research Center for Metabolic Diseases (2020B1111170009). The funders were not involved in research design, data collection and analysis, preparation of the manuscript, or decision to publish.

## Acknowledgments

We are indebted to the participants in this study for their continued and excellent support and our colleagues for their valuable assistance.

## Conflict of interest

The authors declare that the research was conducted in the absence of any commercial or financial relationships that could be construed as a potential conflict of interest.

## Publisher’s note

All claims expressed in this article are solely those of the authors and do not necessarily represent those of their affiliated organizations, or those of the publisher, the editors and the reviewers. Any product that may be evaluated in this article, or claim that may be made by its manufacturer, is not guaranteed or endorsed by the publisher.
