# Supplementary material for: The association of liver enzymes with diabetes mellitus risk in different obesity subgroups: A population-based study
Source: Front Endocrinol (Lausanne). 2022 Oct 13;13:961762. doi: 10.3389/fendo.2022.961762 (PMC9608349; doi:10.3389/fendo.2022.961762)
Supplement: Supplementary file 1 [file Table_1.pdf]

Table S1 Regression model of relationship between liver enzymes and DM risk in common obesity subgroups.

Liver enzymes category[a]	Fold change(95%CI) of DM risk for per unit or class increase in liver enzymes			
	Unadjusted model		Adjusted model[b]	
Central obesity[c]				
AST	0.998(0.97-1.02)	P=0.889	0.97(0.94-1.00)	P=0.093
ALT	1.02(1.00-1.04)	P=0.0322	1.00(0.98-1.03)	P=0.846
GGT	1.04(1.03-1.05)	P<0.001	1.04(1.02-1.06)	**P<0.001**
Non- central obesity				
AST	1.02(0.997-1.04)	P=0.088	0.99(0.97-1.02)	P=0.670
ALT	1.03(1.01- 1.04)	P=0.004	1.01(0.99-1.03)	P=0.537
GGT	1.06(1.05-1.07)	P<0.001	1.05(1.04-1.06)	**P<0.001**
BMI≥28.0 kg/m^2				
AST	1.01(0.96-1.06)	P=0.686	1.01(0.95-1.06)	P=0.798
ALT	1.02(0.99-1.06)	P=0.163	1.01(0.97-1.05)	P=0.721
GGT	1.03(1.01-1.06)	P=0.006	1.04(1.01-1.07)	**P=0.011**
BMI<28.0 kg/m^2				
AST	1.01(0.99- 1.03)	P=0.335	0.98(0.96- 1.00)	P=0.103
ALT	1.03(1.01- 1.04)	P<0.001	1.00(0.99-1.02)	P=0.593
GGT	1.05(1.05-1.06)	P<0.001	1.05(1.04-1.06)	**P<0.001**

[a] linear regression models for liver enzymes;

[b]Covariates in the adjusted model: gender, age, BMI, SBP, TG, HDL.

[c]WC>90cm in male or WC>85cm in female were divided into central obesity.
